# Hypothesis: Towards the origin of cancer epidemics and pathogenesis

**DOI:** 10.4103/1477-3163.61265

**Published:** 2010-03-24

**Authors:** Sergey Rumyantsev

**Affiliations:** Department of Immunology, Andent, Inc. Waukegan, IL 60085, USA

**Keywords:** Cachexia, cancer pathogenesis, hereditary immunity, heterozygosity, metastases

## Abstract

**Background:**

The article presents the initial results of an attempt to reconsider current data about cancer epidemiology and pathogenesis from the viewpoint of recent all-pathological, immunological, genetic and evolutionary discoveries.

**Methods:**

The investigation was based on a multidisciplinary approach to reassessment and reinterpretation of relevant current data about cancer epidemiology, clinical manifestations and course.

**Results:**

In contrast to the current 50-year-old hypothesis of mutant maternal tumor and its subsequent metastasis, the revealed set of evidences allowed hypothesize that potentially cancerous cell clone spreads in human population and settles some persons‘ bodies during cross-fertilization of parents with genetically incongruent regulators of cell dividing and tissue growth. The clone is formed and distributed in the offspring‘s body before postnatal ontogenesis and for many decades exists in it like sleeping populations of smallest sizes. But at a relevant time of an individual‘s life (mainly after 40 years of age), according to a specific program of the clone ontogenesis, the populations come into sight as constitutionally immune against prevailing regulators of cell reproduction and begin to multiple uncontrollably thus initiating the cancerous growth.

**Conclusions:**

The new view of cancer origin and pandemic spread supplies a framework for understanding the genetic nature of cancer epidemics and its rising incidence in the current worldwide population. It also forces one to reconsider the perspective of future investigations and reassess both the means and methods for cancer prevention and healing..

## INTRODUCTION

Cancer has become a disease of greater concern and fear among the public as it places a significant emotional and economic burden on families and governments. Despite considerable epidemiological, immunological, genomic, surgical and pharmacological efforts, the cancer epidemic continues to increasingly take its toll on human life. The current efficacy of cancer prevention and treatment is very low. The closely connected issues of the origin, initial causes, pathogenesis and epidemic spread of the disease are not yet satisfactorily understood. There is a gap in the understanding of events leading to cancer and as such, there are opportunities for novel ideas and concepts to explain the status quo.

For the last 50 years the prevailing paradigm in cancer origin, pathogenesis, prevention and treatment has been based upon the rarely questioned ‘somatic mutation hypothesis’, which states that: (1) cancer is derived from a single somatic cell that has accumulated multiple DNA mutations in genes that control proliferation and the cell cycle, (2) cancer is a disease of cell proliferation that leads to (3) the formation of a maternal tumor and (4) subsequent spread (metastasis) of cancer maternal cells outside of primary site to form daughter tumors in distant locations in the body. The epidemic spread of cancer among human populations is outside of the scope of this hypothesis, a hypothesis which contains many questionable assertions about many of its premises.

The present article aims to initiate a revision of the questionable assertions of contemporary oncology. In contrast to the current paradigm, our preliminary hypothesis was that a potentially cancerous cell clone does not appear in a body as a result of specific mutation of a somatic cell. The clone is of innate origin formed by heterozygous interbreeding. It settles in the human body during yearly ontogenesis and for many decades remains dormant as stochastically distributed microscopic cell populations. But at a relevant time in an individual‘s life (mainly after 40 years of life), the clone responds according to its own program of ontogenesis and comes into sight as constitutionally immune to normal cytoecological regulators and begins its malignant development.

## MATERIALS AND METHODS

The investigation was based on theoretical reassessment and reinterpretation of well-known data about cancer epidemiology, clinical manifestations and course[1] from an integrated viewpoint of recent all-pathological, oncologic, immunological, genetic and evolutionary discoveries.[2] The first step in this new contribution to cancer descent and epidemic spread has been initially supported by the Science Advisory Board publication on www.scienceboard.net.[3] The investigation included and integrated the recently discovered all-pathological, immunological, genetic and evolutionary prerequisites (a) for subsequent reassessment of the unique and ordinary features of cancer disease (b). The preliminary results allowed a model of cancer molecular pathogenesis to be proposed (c) to explain the uniqueness of both cancer genetics and its epidemic spread (d). The main emphasis has been on the genetic predilection to cancer and the origin of distribution of cancer affections amongst different human populations, individuals and especially among different parts of a diseased body.

## RESULTS AND DISCUSSION

### a) All-pathological prerequisites for the revision

Any disease displays a set of universal signs that are also characteristic of other diseases. Each of these universal features illustrate the all-pathological phenomenon of heterozygous mosaicism created by genetic admixture arising as a result of heterozygous sexual hybridization between two genetically different organisms: one of which is constitutionally immune to the relevant ecological or physiological agent whereas itsmating partner is constitutionally sensitive to it.[2]

**Figure 1:**
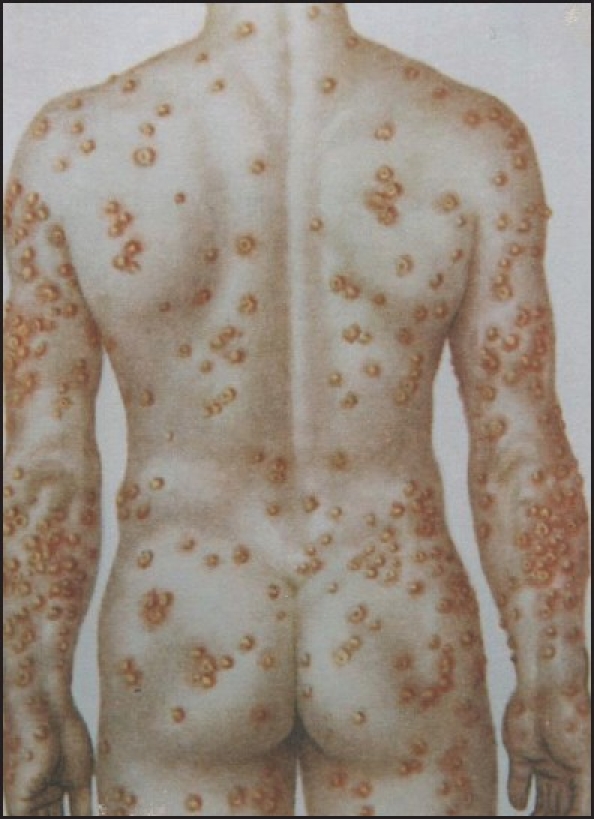
Mosaic spottedness of infectious damages: mutual arrangement of hereditary immune and susceptible loci of the skin tissue in a case of smallpox.

**Figure 2:**
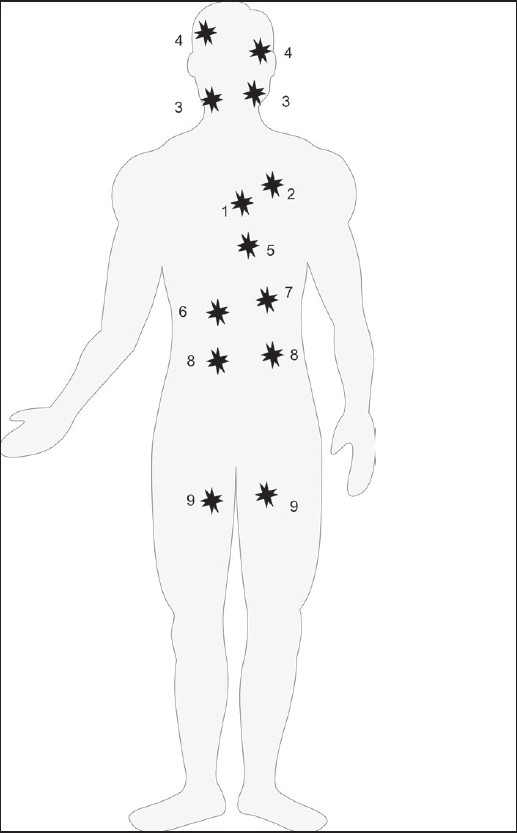
Most dangerous locations of atherosclerotic affections: 1) coronary artery (coronary arterial disease) 2) aorta (aortic aneurysm) 3) internal carotids (stroke) 4) cerebral arteries (stroke) 5) abdominal aorta (aortic aneurysm) 6) hepatic artery (insufficiency of liver), 7) pancreatic artery (insufficiency of the pancreas), 8) renal arteries (kidney ischemia, a silent killer disease), 9) femoral arteries (peripheral arterial disease).

The heterozygosity results in the coexistence of at least two active allelomorphic genes in the offspring’s genome. Both alleles function dominantly and create two allelic cell clones whose subpopulations are formed and distributed in the body before postnatal ontogenesis. The heterozygous offspring expresses both alleles equally but in different sizes and separated locations around the body [Figures 1 and 2]. The features and functions of codominant clones may become obvious at different steps of ontogenesis.[4]

Genetic admixture (also called xenogamy, outbreeding, crossfertilization, crossbreeding) refers to the reproductive union of genetically dissimilar or unrelated organisms within the same species that inevitably results in offspring heterozygosity of various kinds. The states of heterozygosity are responsible for the origin of spotted mosaic manifestations, individually different course and severity of most diseases, both infectious and non-infectious.[2]

The mosaicism is revealed in genetically determined variations in the location, size and other pathological manifestation of any disease. Every human disease is extraordinarily diverse in its manifestation. Affected people have many individual differences in the manifestations of their illnesses as well as in the grade of expression. The shape, location, size and rate of cancer progression are also very different in different individuals.

The set of universal all-pathological features includes at least a dozen intrinsic signs: a) different incidence of a disease among different races and ethnic groups, b) increased prevalence of diseases in developed and civilized countries, c) genetic predilection to the disease, d) age differences in the disease incidence, e) stochastic distribution of individual cases amongst a population, f) individual variations in constitutional (genetic) predilection to the disease, g) the mosaicism of affections, i.e. intra-individual diversity both in the predilection of different parts of a tissue and in the quantity and sizes of affections, h) dappled distribution of affections amongst a body, i) molecular bases of genomic and cellular pathogenesis and j) the identity of involved cells in any locations of specific affections around the body.[2] Each of these universal signs of pathology belong to any form of cancer too. This set of all-pathological data allows us to hypothesize that a potentially cancerous cell clone may also appear in a human body as a result of genetic admixture and then functions according to its own program of ontogenesis.

Recent data about the involvement of inherent immunity in disease determination i.e. molecular steps of diseases’ pathogenesis provides especially supportive evidence of this new view of cancer origin. A deficiency of production of a particular hormone leads to the dysfunction of the relevant metabolic system. The same result can be achieved by mutant modifications of either the hormone or its receptor to form an incongruence between the co-actors, i.e. constitutional immunity against hormone influence.[5–7] The blocking effect of mutant modifications of either hormones or their receptors leads to many pathological states including obesity.[8] Genetic immunity of cells to insulin is a major determinant of the decline of glucose tolerance. Non-insulindependent diabetes mellitus is characterized by pathological hyperglycemia in the presence of higher-than-normal levels of plasma insulin. A pathogenic decrease in cell sensitivity to vitamin D3 determines the familiar forms of rachitis. The immunity of cells to androgens causes the phenomenon of testicular feminization. The resistance of cells to corticosteroids determines the pathogenesis of Cushing’s disease.[8] The grade of the cells’ immunity to thyroid hormone determines the range of relevant disturbances. This resistance is an inherited inability to respond appropriately to the T3 hormone linked to mutations in the thyroid hormone receptor (TR)-beta[9] The principles of cell immunity to physiological agents are analogous to those in constitutional (genetic) immunity to infections.[2] One can hypothesize an analogous origin of cancer cell immunity against incongruent molecular physiological regulators of cell division and growth. This supposition, together with mutual exposure, analysis and evolutionary comprehension of a set of relevant immunological data, allowed us to put forward a new idea about the molecular pathogenesis of cancer.

### b) The uniqueness of cancer disease

Each of the universal signs of any pathological state is also consistent with any form of cancer, however, the origin and development of malignancy reveals some unique traits. Firstly, in contrast to any other disease, cancer comes into sight when the division and growth of some cells in some parts of the body become uncontrolled. The disturbance can be explained by the resistance i.e. constitutional (genetic) immunity of the involved cells to relevant molecular physiological regulators. Secondly, whereas the cells’ resistance to hormonal or infectious influences has no visible distinctions from the susceptible ones, the cancer cells look abnormal under the conventional light microscope. They are considered versions of cells which compose the tissue of the supposed cancer origin, however, light microscopy cannot identify the tissue and site of a malignancy origin.[10] Thirdly, cancer genetics holds some mystery which should be taken into account.

Thus cancer is more ordinary than it is unique. Its uniqueness is the abnormality of its cell morphology and aggressive behavior provided by uncontrollable division and growth. The molecular basis of the genomic and cellular pathogenesis of cancer cannot therefore be considered as principally unique because they are associated with the constitutional immunity against molecular physiological regulators which are responsible for many non-cancerous diseases.[8] This kind of autoimmunity is determined by constitutional incongruence between relevant physiological regulators and its receptors. The set of above data provides an explanation of the most unique feature of cancer, its aggressive behavior provided by uncontrollable division and growth, and thus allows us to propose an alternate explanation of cancer pathogenesis.

### c) The proposed molecular pathogenesis of cancer

The (distinct) distribution of affections is the principal feature of any disease including cancer. That might mean that within any body affected by cancer there are initially two coexisting cell clones of similar origin with opposite predisposition to the development of malignancy. The potentially cancerous clone exists in a form of distantly separated populations and their initial sizes are different but very small. They are dispersed around the body in a manner that is not yet understood, for example, like congenital small dark spots on the human skin known as moles, melanocytic nevuses exist in a form of benign neoplasm but may pose a higher risk of malignant melanoma.[11] Because the allelic clone is not eliminated by individual adaptive immunity it is not alien to the lymphatic system. This means that both the emergence of the cancerous clone and the dispersion of its subpopulations around the body could been performed before postnatal ontogeny.[12]

The genetic but not antigenic ‘alienness’ of the cancerous clone is also evidenced, for instance, by the unexpected link between stomach cancer and a type of undifferentiated stem cell that originates in the bone marrow.[13] Analogically, stem cells, not ordinary somatic cells cause breast cancer. Beside, only a tiny minority of cells in these human tumors is capable of inducing experimental cancers; the rest are relatively harmless.[14] These observations may also evidence the antenatal origin of cancerous clones.

Different locations of potentially cancerous clone subpopulations appear visually detectable in different times after initiation of the clone’s malignant growth that allow the supposition of differences in their initial sizes. The differences both in initial cancer cell micromasses and in their dislocation around the body predestine individual diversity in the course and severity of cancer when the disease begins its malignant development.

Most cells of a cancer clone are mainly located in the organ or tissue which is considered the place of their origin. Other subpopulations of the same clone are distantly located. Beside, some patients may have a cancer whose site of origin is hidden and never identified.[10] Various organs can serve as the places for distant origins of cancer (the lungs, liver, brain, bones *et al*.). In contrast, the populations of malignant cells that form leukemia, multiple myeloma, and lymphoma are not localized but dispersed around the affected body like prevailed cell clones of the same tissue, and relevant cancer cells may be found in the blood, several lymph nodes, or other parts of the body such as the liver and/or bones.[1]

All distant locations of a cancer consist of identical cells. This feature is paradigmatically considered as a result of metastasis. [1] According to the paradigm, cancer cells metastasize mainly through the bloodstream or the lymphatic system although the existence of the process has not been evidenced. In reality we can only observe the non-simultaneous appearance of several identical tumors in different parts of a diseased body. The details of the metastasis remain hypothesized and mysterious.[15] In contrast to this paradigm, distant cell distribution can be considered a result of the well-known phenomenon of cell translocation during embryogenesis. Translocations can result in serious congenital disorders like dysplasia, sarcomas, leukemia, *et al*.

The “metastatic ability” of cancerous cells is considered a late event in the development of malignant disease. Meanwhile, it has been shown that potentially cancerous cells may establish residence in different locations in the body and may exist there long before they begin to have malignant growth. Beside, such cells can survive in unusual (ectopic) sites for an extended period of time and proliferate only after oncogene activation.[16,17] Such observations confirm the above supposition about own program of ontogeny possessed by cancerous clones.

For many of the initial decades of an individual’s life the potentially cancerous cell clone exists in the body in a state of reproductive lethargy. But at a relevant time (mainly after 40 years of life), probably according to a specific program of this cell population’s ontogenesis, the clone begins its malignant development. The phenomenon of diversity in the cell clone’s programs of ontogenesis has been mentioned, for instance, at the genesis of aging.[18]

Four types of different cancer progression have been revealed by screening observations performed over 20 years on breast and prostate cancers from the time the first tumor cells appear while the tumor is not yet detectable (microscopic); when it can be detected as belonged to the organ; regional (when other localizations appear); and to the point when previously least cell populations appear as “metastases” and death occurs.[19] Tumors Type A are the least aggressive and remain undetectable and without morbidity during the patient lifetime. Tumors Type B grow until they become detectable but never cause symptoms and lead to death. Tumors C are programmed to become “metastatic” and fatal. Tumors D are the most aggressive. They also are destined to become “metastatic” and grow extremely quickly. By the time they can be detected they may no longer be curable.

Before or at the time of the commencement of malignity, the cells come into sight as susceptible to either inside or ecological carcinogens, for instance to infections causing human cancer[20] yet are constitutionally resistant to regulators of both cell reproduction and the growth of cell populations inside. Constitutional (hereditary) immunity of the clone against relevant physiological regulators can be created by structural incongruence between regulators and their receptors. The existence of such specific immunity should be considered as the obligate prerequisite to malignity. Besides, the uncontrollably growing populations of a cancerous clone could produce its own regulator which may suppress the functions of normal cells thus inducing the development of cachexia.[21]

Cancer cachexia is mainly characterized by extensive loss of adipose tissue and skeletal muscle. The loss of skeletal muscle mass is especially characteristic of cancer. Cachexia is thought to be induced by some circulating tumor factor that has a direct catabolic effect on host tissues, such as lipid mobilizing factor, which acts on adipose tissue, and proteolysis-inducing factor, which acts on skeletal muscle. Loss of adipose tissue appears to be due to an increase in the degradation of triglycerides, rather than a decrease in synthesis.[21] Paradoxically, the cachexia syndrome occurs only in about half of all cancer patients. One can suppose that skeletal muscle and adipose tissues of the other half of patients are resistant to the cachexia-inducing agents produced by the tumor.

### d) The oddities of cancer genetics

A series of recent genetic investigations confronted the somatic mutation theory with a number of apparent paradoxes and alternative views began to be articulated.[22] For instance, the malignant phenotype is determined largely by early transforming events rather than being molded by somatic evolution during the clonal expansion of neoplastic cells.[23–25] Recent data of cancer genome sequencing show that almost all the changes in the gene structure are heterozygous and present in nearly all the cells in the discovered tumor samples.[26]

In addition to these recent observations, there is a well-known fact that the vast diversity of normal cell phenotypes in any living body is generated by the same genome. What is more, the initiation of cancer is influenced by the inherited cancer-promoting genotype.[16,17] Such observations prompt bench scientists to reason whether somatic mutation and selection are really necessary and sufficient to produce the sophisticated survival skills of invading and disseminated malignant cells.[27,28]

Although it is now a confirmed fact that a person’s genetic makeup has a principal influence on the development of cancer, nothing is known about the special characteristics of the genome that determine the unregulated behavior of cancer cells and their spotted distribution around the body. In contrast, the genetic origin of analogous distinct distribution in both infectious and many noninfectious diseases as well as the role of constitutional immunity in their molecular pathogenesis has been deciphered recently by many investigators.[2]

The phenomenon of genetic predilection to cancer is an important source of doubt both in the somatic mutation hypothesis and in the existence of metastasis. The predilection is predetermined by a set of genetic factors associated mainly with genetic admixture and with the genesis of aging.[18] The cancerous insertion in the genome could happen at the early stages of anthropogenesis as a result of genetic admixture.

This kind of genome transformation has especially influenced anthropogenesis.[2,29] Now, the special genetic program established in a human cancerous cell clone is highly preserved in its distant locations.[25] Because it begins to function at the end of reproductive age, this highly pathogenic insertion has not been eliminated by natural selection.

Cancer rates in the Californian population of South Asians, that comprise people having origins mainly in India, Pakistan, Bangladesh and Sri Lanka, are different from those breast cancer.observed in other ethnic groups inhabiting the same state. Compared to rates in native Asian Indians, rates of cancer in South Asians of California were higher for all sites except oropharyngeal, esophageal and cervical cancers. Compared to Asian/Pacific Islanders of California, the South Asian population experienced more cancers of the esophagus, gall bladder, prostate, breast, ovary and uterus, as well as lymphomas, leukemias and multiple myelomas. Compared to the non-Hispanic White population of California, South Asians experienced more cancers of the stomach, liver and bile duct, gall bladder, cervix and multiple myelomas. Significantly increasing time trends were observed in colon and breast cancer incidence.[30] African-American women have a lower overall incidence of breast cancer than do Caucasian women, but a higher overall mortality and the differences between their breast cancer cell lines play a role in their different rates of cancer metastasis.[31]

The currently observed increasing incidence of most diseases depends on the intensity of the population’s genetic admixture within ethnically mixed populations.[1] The role of xenogamy in the origin, individual manifestations and course of malignant diseases is also evidenced by a plethora of epidemiological and clinical observations and investigations. Very mixed African- Americans are more likely to die from cancer then any other racial or ethnic population. In contrast, Hispanics, Asian Americans and Pacific Islanders have lower incidence rates than Whites for the most common cancers. The frequency of colorectal cancer varies around the world. This kind of malignancy is common in the Western world and is rare in Asia and Africa.[1] Although only one cancerous clone usually exists in an affected body, the presence of a number of cancerous clones has also been documented. In a population of a developed country with high survival rates, multiple cancers often comprise two or more primary cancers occurring in an individual that originate in a primary site or tissue and are neither an extension, nor a recurrence or metastasis.[32]

Cancer patients have a 20% higher risk of a new primary cancer compared with the general population.[1] As the numbers of cancer survivors and of older people increases, occurrence of multiple primary cancers is also likely to increase.[32–36] Approximately one-third of cancer survivors aged >60 years were diagnosed more than once with another cancer. Possibly, these variations are associated with the phenomenon of clonal diversity in the genetic programs of the progression of senescence.[18] Such observations prompt the idea of the possible existence of a few potentially cancerous clones in the body.

The exposure, analysis and evolutionary comprehension of epidemiological, clinical, immunological, genetic and experimental data concerning the principal characteristics of cancer showed that many factors are also common to other kinds of diseases. These results provide a radically different view of how cancer tumors come into existence. They allow the proposal that cancer development is determined by a constitutional immune incongruence between normal molecular cyto-ecological regulators and their molecular targets on the cells of potentially cancerous clones. These features of individual molecular constitution exist in a population as a result of genetic admixture between people evolved in ecologically distinct environments and gain differences in their molecular constitution on the level of the regulators of cell growth, development and differentiation.

Individual and intra-individual diversity in cancer course, including the manifestations and severity of specific affections, their sizes and focal disposition around the body, could be created by inter-ethnic mating of persons with incongruent regulator-receptor systems. In the past, it was naturally assumed that cancer was caused by the transformation of normal cells into malignant cells. In contrast, according to the hypothesis formulated and examined above, the genesis of cancer is primordial and its spread in the human population is associated with both, evolutionary formation of interethnic differences and genetic admixture of the offspring with specifically incongruent cell clones. This statement may change the way we view the origin of cancer.

The achievement of the new knowledge about cancer origin, immunopathogenesis, individual course and epidemic spread supplies a framework for understanding the epidemic nature of cancer and its rising incidence both in the developed world and in developing countries. It also forces the reconsideration of the perspectives of future investigations and the reassessment of both the means and methods for cancer prevention and treatment. For instance, it highlights both the possibility of genetic methods for the prevention of the epidemic spread of the disease and the doubts the existence of metastatic processes. Control of genetic admixture could be most effective way to prevent the growth of cancer incidence. But before new preventive perspectives can be realized, tests to determine the danger of cancerous genetic admixture should be explored.

## EDITOR’S NOTE

This manuscript is not peer reviewed. In keeping with the spirit of the journal that likes to provide an opportunity for cancer researchers to share their hypothesis and ideas, which might be out of the vogue. Therefore, this publication should be considered as the opinion of the authors and open for discussion and comments from the readers.

Editors

Journal of Carcinogenesis
